# In Situ Soft X-ray Spectromicroscopy of Early Tricalcium Silicate Hydration

**DOI:** 10.3390/ma9120976

**Published:** 2016-12-01

**Authors:** Sungchul Bae, Manabu Kanematsu, Daniel Hernández-Cruz, Juhyuk Moon, David Kilcoyne, Paulo J. M. Monteiro

**Affiliations:** 1 Department of Architectural Engineering, Hanyang University, Seoul 04763, Korea; sbae@hanyang.ac.kr; 2 Faculty of Science and Technology, Tokyo University of Science, 2641 Yamazaki, Noda, Chiba 278-8510, Japan; manabu@rs.noda.tus.ac.jp; 3 Faculty of Engineering, Universidad Autónoma de Chiapas, Tuxtla Gutiérrez, Chiapas 29050, Mexico; dhernandezcruz@gmail.com; 4 Department of Civil & Environmental Engineering, National University of Singapore, 1 Engineering Drive 2, Singapore 117576, Singapore; 5 Lawrence Berkeley National Laboratory, 1 Cyclotron Road, Berkeley, CA 94720, USA; ALKilcoyne@lbl.gov; 6 Department of Civil and Environmental Engineering, University of California, Berkeley, CA 94720, USA; monteiro@berkeley.edu

**Keywords:** hydration, tricalcium silicate, C–S–H, kinetics, spectromicroscopy

## Abstract

The understanding and control of early hydration of tricalcium silicate (C_3_S) is of great importance to cement science and concrete technology. However, traditional characterization methods are incapable of providing morphological and spectroscopic information about in situ hydration at the nanoscale. Using soft X-ray spectromicroscopy, we report the changes in morphology and molecular structure of C_3_S at an early stage of hydration. In situ C_3_S hydration in a wet cell, beginning with induction (~1 h) and acceleration (~4 h) periods of up to ~8 h, was studied and compared with ex situ measurements in the deceleration period after 15 h of curing. Analysis of the near-edge X-ray absorption fine structure showed that the Ca binding energy and energy splitting of C_3_S changed rapidly in the early age of hydration and exhibited values similar to calcium silicate hydrate (C–S–H). The formation of C–S–H nanoseeds in the C_3_S solution and the development of a fibrillar C–S–H morphology on the C_3_S surface were visualized. Following this, silicate polymerization accompanied by C–S–H precipitation produced chemical shifts in the peaks of the main Si K edge and in multiple scattering. However, the silicate polymerization process did not significantly affect the Ca binding energy of C–S–H.

## 1. Introduction

Tricalcium silicate (C_3_S, C=CaO, S=SiO_2_) is well-known as the main component of the Portland Cement (PC) clinker [1]. Its hydration reaction, which is exothermic, produces calcium silicate hydrates (C–S–H) and calcium hydroxide (Ca(OH)_2_, CH) [2]. Early hydration of C_3_S in water occurs by the dissolution of C_3_S and precipitation of C–S–H and CH. C–S–H is the primary hydration product of C_3_S and has a marked influence on the mechanical properties and long-term durability of PC-based materials [3,4]. C–S–H exhibits only short-range crystallinity, which is the most prominent physicochemical factor controlling mechanical and chemical properties in the production of PC-based materials. The kinetics of C–S–H precipitation during the first hours of C_3_S hydration have been of great interest in cement chemistry because of their immense impact on the setting time and hardening of concrete [5,6].

There are four periods in early hydration of triclinic C_3_S: (a) initial reaction; (b) induction; (c) acceleration; and (d) deceleration [2,5]. In the period of slow dissolution, the induction or dormant period, the rate of C_3_S hydration decreases within the first few minutes and then remains low [7]. In the case of pure C_3_S hydration, this dormant hydration period is generally very short [8]. Geochemical theory and the formation of an inhibiting or protective layer have been proposed to explain the experimental results of C_3_S hydration kinetics in the induction period [7,9]. While suggested models for explaining the mechanism governing C_3_S hydration kinetics remain a topic of debate, hydration is generally recognized to be a process of dissolution and precipitation [7,8]. When mixed with a limited amount of water, C_3_S dissolves readily and C–S–H nucleates on its surface within minutes; the C–S–H starts to grow rapidly at the end of the induction period. In this process of dissolution and precipitation, the silicate monomer is dissolved from C_3_S and defective sheets of tetrahedral SiO_4_^4−^ are formed through silicate polymerization. The molar ratio of Ca to Si (Ca/Si) and the chain length of C–S–H change correspondingly due to silicate polymerization [8,10]. The growth rate of C–S–H has been shown to depend on the mechanism of C_3_S hydration kinetics in the acceleration period. Although an understanding of the mechanism of the C_3_S hydration process is vital in view of its function as the major component of the PC clinker, many issues related to the induction period, such as the mechanism of initiation and end of the period, are still under contention [7].

The early age C_3_S hydration process has been studied by many characterization techniques, including calorimetry [8,11,12,13], in situ X-ray diffraction [14], X-ray photoelectron spectroscopy (XPS) [10], electron microscopy [1,15], and in situ neutron scattering [6]. However, the methods described above are used for bulk analysis and are limited when providing local morphological and atomic-binding structural information about the hydration products. While traditional electron microscopy has provided morphological details of cement hydrates, its operation requires a high vacuum environment, resulting in morphological changes to the cement hydrates [3,16]. Recently, new in situ techniques for imaging morphology and mapping chemical composition of wet samples [17,18], such as environmental scanning electron microscopy (ESEM) and synchrotron hard X-ray nanoprobes, have been used to study cement hydration. However, none of these can provide nanoscale spectroscopic information about the various hydration products as hydration proceeds.

Soft X-ray spectromicroscopy can provide a new perspective of the hydration process because it allows the measurement of both near-edge X-ray absorption fine structure (NEXAFS) spectra and X-ray absorption images, with high image and spectral resolutions of 30 nm and 0.1 eV, respectively [19]. The scanning transmission X-ray microscopy (STXM) technique has been successfully employed in numerous applications [20,21,22], including cement chemistry [23,24,25]. Recently, in situ soft X-ray spectromicroscopy using wet cells created by sandwiching a sample between two silicon nitride (Si_3_N_4_) windows has been widely utilized for investigating wet samples [26,27,28]. Although this in situ X-ray spectromicroscopy technique employing wet cells has great potential for studies of cement hydration, it has limited applications for studying PC hydration. This is due to insufficient availability of NEXAFS data of the cement clinker phases and hydration products. In particular, although Si and Ca are the main elements in PC hydration products, Ca L_III,II_ and Si K edge NEXAFS spectra of cement hydrates are extremely limited. In previous works, we demonstrated the sensitivity of NEXAFS spectra to the local structure and valence, which can be used to study the local binding structure of C–S–H [19,25]. NEXAFS analysis of synthetic C–S–H at different Ca/Si ratios clearly shows that enhanced silicate polymerization of silicate tetrahedra corresponds to a higher peak position of the Si K edge as well as that resulting from multiple scattering [25]. An identical trend in chemical shifts was also observed in the NEXAFS study using C_3_S paste hydrated for 17 days [25]. In the early age C_3_S hydration process, dissolution of the tetrahedral SiO_4_^4−^ monomer from C_3_S and the formation of C–S–H are expected to affect both the morphology and the Ca and Si binding energies of C–S–H. However, this has not been thoroughly investigated. This investigation is one of the objectives of this study.

Here, we present the first in situ soft X-ray spectromicroscopy of the early age of the C_3_S hydration process. For direct in situ observation of C_3_S hydration over time, C_3_S paste was cast and placed in a wet cell. The evolution of microstructure through a dissolution-precipitation process and its effect on the binding energy of C–S–H were investigated by X-ray absorption imaging and Ca L_III,II_ and Si K edge NEXAFS analysis from the end of the induction period to the deceleration period in the hydration. This new approach to studying the kinetics of C_3_S hydration provides a spectroscopic explanation of the hydration process of C_3_S and sheds light on the early age of the C_3_S hydration mechanism.

## 2. Materials and Methods

### 2.1. Wet Cell Production for In Situ STXM Measurement

**Figure 1:**
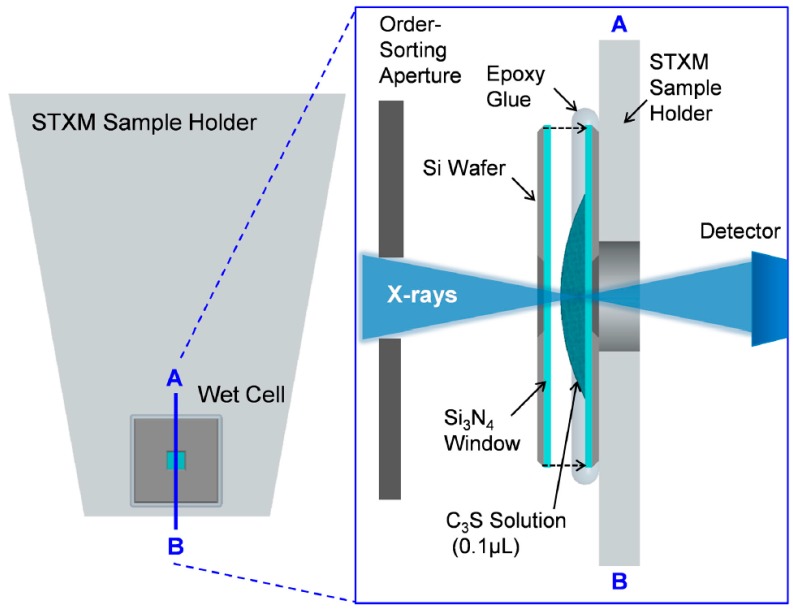
Schematic representation of the STXM (Scanning Transmission X-ray Microscopy) sample holder and cross-sectional overview of the wet cell. Transmitted X-rays are detected by a single-element detector, and the image is obtained from the detector signal as a function of the sample position.

Powdered pure triclinic C_3_S was obtained from the commercial suppliers CTLGroup (Skokie, IL, USA). Freshly ground C_3_S was mixed with deionized water to a water-to-C_3_S ratio of 5:1 to disperse the particles. Wet cells using a C_3_S solution for in situ measurement (from ~1 to 8 h) were produced as illustrated in Figure 1. A 0.1 μL drop of the C_3_S solution was taken and placed on the center of the Si_3_N_4_ membrane window (window size: 1 × 1 mm, membrane thickness: 50 nm, and silicon wafer size: 5 × 5 mm), and another membrane was placed on top of it. Epoxy glue was employed to seal the two membrane windows to minimize the evaporation of moisture inside the wet cell. The residual C_3_S solution was kept in a falcon tube with N_2_ gas at 23 °C and sealed in a vacuum bag. The C_3_S solution was kept in a carbon-free chamber and utilized for later-age ex situ measurements (after 8 h–40 days). For measurements at a later age, the C_3_S solutions placed on a single Si_3_N_4_ membrane window were used. To avoid possible beam damage of the samples due to continuous X-ray irradiation, different particles from the same solution were used for each measurement. Before loading the wet cell into the STXM chamber, an optical microscope was used to detect the presence of cracks on the membrane window of the wet cell. Helium gas was simultaneously injected into the STXM chamber while the residual gas in the chamber was pumped out to prevent cracking of the wet cell. Beam damage to the sample from X-ray radiation was assessed by checking for morphological and spectroscopic changes within specific areas before and after each measurement. No evidence of radiation damage to the sample was found in the current study.

### 2.2. Scanning Transmission X-ray Microscopy (STXM)

Soft X-ray spectromicroscopy data was collected by means of STXM at the Advanced Light Source (ALS) branch line 5.3.2.1 and 5.3.2.2, with a synchrotron storage ring operating at 1.9 GeV and 500 mA of stored current. Counting times of the order of a few milliseconds or less per pixel were used in order to avoid beam damage. X-ray absorption images and NEXAFS spectra around the Ca L_III,II_ edge (340–360 eV) and Si K-edge (1825–1890 eV) were measured on beamline 5.3.2.2 and 5.3.2.1, respectively, due to the different energy ranges (e.g., STXM 5.3.2.1: 500–2500 eV, 5.3.2.2: 250–800 eV). The spectral and imaging resolutions were ±0.1 eV and 30 nm, respectively. To calibrate the energy positions, CaCO_3_ and quartz (SiO_2_) were used as references for the Ca L_III,II_ and Si K edges, respectively.

### 2.3. Isothermal Calorimetry

Heat flow in the C_3_S paste at an early age was monitored in an isothermal conduction calorimeter (TAM-air, TA instruments, New Castle, DE, USA) at a constant temperature of 23 °C. One gram of C_3_S was prepared, with a water-to-C_3_S mass ratio of 5:1. Distilled water and C_3_S powder were hand-mixed inside a glass ampule for 2 min. The paste was tightly sealed immediately after mixing and placed in an isothermal calorimeter. Frictional heat due to the introduction of the sample (~10 min) was not included in the analysis.

## 3. Results and Discussion

### 3.1. Isothermal Calorimetry

**Figure 2:**
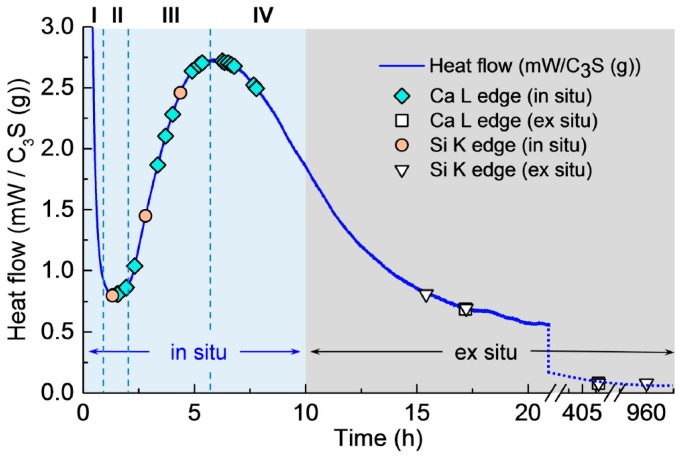
Isothermal calorimetry curve for hydration of C_3_S paste (W/C_3_S = 5) at 23 °C.

Isothermal calorimetry was conducted on the C_3_S paste to determine the hydration periods in the in situ STXM measurements (see Figure 2). The isothermal calorimetry curve of the C_3_S paste exhibited the four expected hydration periods: (I) dissolution (0–1 h); (II) induction (approximately 1 h); (III) acceleration (approximately 1–6 h); and (IV) deceleration (after 6 h), in good agreement with previous studies [7,8,11,29]. The symbols in the figure identify the measurement times of in situ and ex situ STXM.

### 3.2. In Situ STXM at an Early Age of C_3_S Hydration

The first in situ STXM of C_3_S paste in a wet cell was recorded 1 h 34 min after mixing with water. This gap between the injection of water and the first measurement includes the time for wet cell production—i.e., epoxy glue hardening (~40 min)—slow application of vacuum for the purpose of preventing cracks in the wet cell (~30 min) in the STXM chamber, and time for scanning and focusing on a particle in the wet cell (~10 min). As illustrated in Figure 2, the Ca L_III,II_, Si K edge NEXAFS spectra and X-ray absorption images were obtained from 1 h 30 min (end of induction period) to 7 h 47 min (acceleration period). After 8 h of hydration (from the deceleration period), ex situ STXM (the end of deceleration period) was performed using the hydrated C_3_S on a single Si_3_N_4_ window.

#### 3.2.1. Morphological Observation of the C_3_S Hydration Process (Ca L_III,II_ Edge)

**Figure 3:**
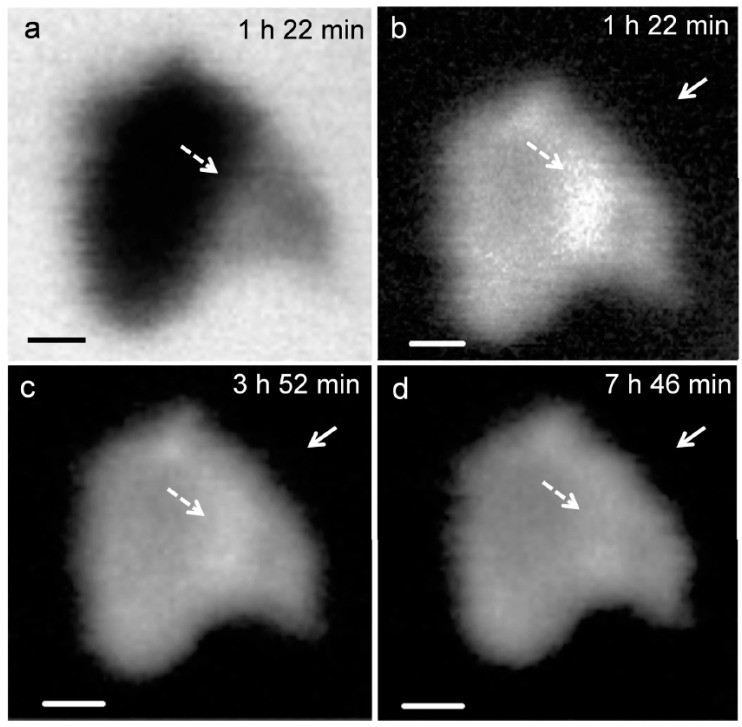
X-ray absorption image taken at 347.2 eV (**a**) in situ morphology of a C_3_S particle in the wet cell from (**b**) 1 h 22 min; (**c**) 3 h 52 min; and (**d**) 7 h 46 min. Scale bar: 200 nm.

Figure 3 presents the in situ STXM image and contrast map of C_3_S in the process of hydration from 1 h 22 min to 7 h 46 min. According to the isothermal calorimetry results (Figure 2), the duration of measurement corresponds to the onset of the period of acceleration and deceleration of C_3_S hydration. Since the low energy for Ca (340 eV) results in low X-ray photon penetration, a relatively small particle was selected for the image contrast map. In the X-ray absorption image (Figure 3a), the darker area represents thicker zones in the C_3_S particle. To obtain the map, images taken at the Ca L_III,II_ edge (349 eV) were subtracted from the same images taken at the Ca L_III,II_ pre-edge [25]. At 1 h 22 min (Figure 3b), the bright white areas, indicated by the dashed arrows, correspond to locations with high concentrations of Ca in the C_3_S particles. Note that the image contrast map only differentiates Ca concentrations or the thickness of a sample but not the chemical speciation. The dissolution of C_3_S was found to occur in the acceleration period, in the localized area (dashed arrow) on the C_3_S particle, which could be related to the surface area or crystalline defects in C_3_S [30]. The localized dissolution area appeared to be the edge of C_3_S surface, as identified in the X-ray absorption image (Figure 3a). After 3 h 52 min, the brightness of the localized dissolution area decreased due to the precipitation of C–S–H on the C_3_S particle indicating that Ca was distributed uniformly on the surface of C_3_S. In Figure 3b, Ca-containing nanoparticles were observed in the solution around the C_3_S particle and are indicated by the straight-line arrows in the images. Agglomeration of the nanoparticles with C_3_S was observed over time, with the C_3_S surface evolving to exhibit a fibrillar morphology. We therefore propose that the nanoparticles found in this study could be C–S–H seeds formed in the C_3_S solution within the first few hours of hydration.

#### 3.2.2. Ca L_III,II_ Edge NEXAFS Analysis

**Figure 4:**
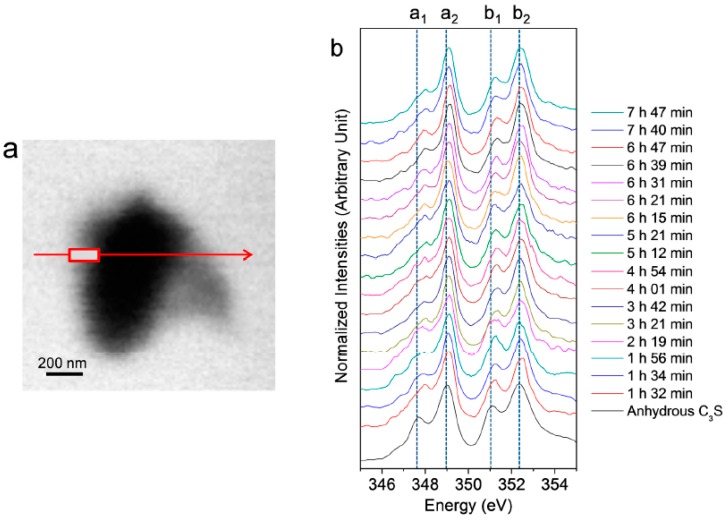
(**a**) Image of a hydrated C_3_S particle in the wet cell (taken at 347.2 eV) and (**b**) Ca L_III,II_ edge NEXAFS (near-edge X-ray absorption fine structure) spectra of the line scan of C_3_S in the wet cell using in situ STXM from 1 h 32 min to 7 h 47 min after mixing with water.

In situ Ca L_III,II_ edge NEXAFS spectra were acquired on the C_3_S particle in the wet cell from 1 h 32 min to 7 h 42 min, as shown in Figure 4. The reference spectrum of the anhydrous C_3_S powder and Ca(OH)_2_ is from our previous work [25]. In the present study, two different modes—line scan and image stack—were used for the in situ and ex situ NEXAFS measurements, respectively. The NEXAFS spectra using continuous X-ray absorption images, called an image stack, show better absorption features with lower background noise compared to the line scan. However, acquisition of the image stack for the Ca L_III,II_ edge NEXAFS requires at least 40 min. Therefore, there is a possibility that chemical changes may occur during the measurement. Moreover, the core and right area of the C_3_S particle were not thin enough to absorb sufficient photons of the transmitted X-ray beam, resulting in X-ray beam saturation. Therefore, we conducted a line scan for the in situ Ca L_III,II_ edge NEXAFS analysis. The area (rectangle) in the line scan (arrow) was selected for the analysis since it requires a significantly shorter measurement time (~3 min) compared to the image stack. Each spectrum was normalized to the intensity of the incident beam, I_0_, after removal of the linear pre-edge background.

As shown in Figure 4, the Ca L_III,II_ edge NEXAFS of the Ca compounds consists of two main spin-orbit-related peaks, L_3_ 2P_3/2_ (a_2_) (electronic excitation 2p^6^3d^0^ → 2p^5^3d^1^) and L_2_ 2P_1/2_ (b_2_) (2p^6^3d^0^ → 2p^5^4s^1^), along with a number of smaller peaks, a_1_ and b_1_, that precede the main peaks [31,32]. Generally, these multi-peak patterns are induced by the crystal field, which arises due to the symmetry of the atoms surrounding the Ca cation in the first coordination sphere [31]. The peak positions and energy splitting values of the Ca L_III_ and L_II_ edges of the selected area of the C_3_S surface from 1 h 32 min to 7 h 47 min are given in Table 1. After 1 h 32 min, peaks a_1_ and b_1_ had shifted by 0.3 and 0.2 eV, respectively, to higher energy levels as compared to the NEXAFS spectra of the anhydrous C_3_S, and they showed similar peak positions as the synthesized C–S–H investigated in a previous work [25]. The smaller energy splitting arises from the lower degree of crystallinity of C–S–H compared to anhydrous C_3_S. From 1 h 56 min to 7 h 47 min, there was no significant variation in the peak positions of the Ca L_III,II_ edge of the selected area, fluctuating within ±0.1 eV. As shown in Table 1, Ca(OH)_2_ exhibits larger splitting energy values between a_2_−a_1_ and b_2_−b_1_ compared to anhydrous C_3_S and C–S–H, which is an indication of the well-developed crystalline Ca structure [33]. Based on the peak positions and energy splitting values of the Ca L_III,II_ edges of anhydrous C_3_S, Ca(OH)_2_ and synthetic C–S–H, it can be concluded that the area (surface of C_3_S) selected for analysis mostly consists of C–S–H, not anhydrous C_3_S or Ca(OH)_2_. Note that the peak position and energy splitting values of the Ca L_III,II_ edge NEXAFS spectra for the selected area, mostly C–S–H, showed no significant changes during the hydration process, which is in good agreement with the previous findings that the synthesized C–S–H with a Ca/Si ratio of 0.66 to 1.44 showed no variation in terms of peak positions and splitting energies for Ca L_III,II_ [25]. This trend was also confirmed in a previous study of Ca K edge X-ray absorption spectra of C–S–H, indicating that silicate polymerization does not appear to affect the Ca K edge absorption feature of C–S–H [34].

**Figure 5:**
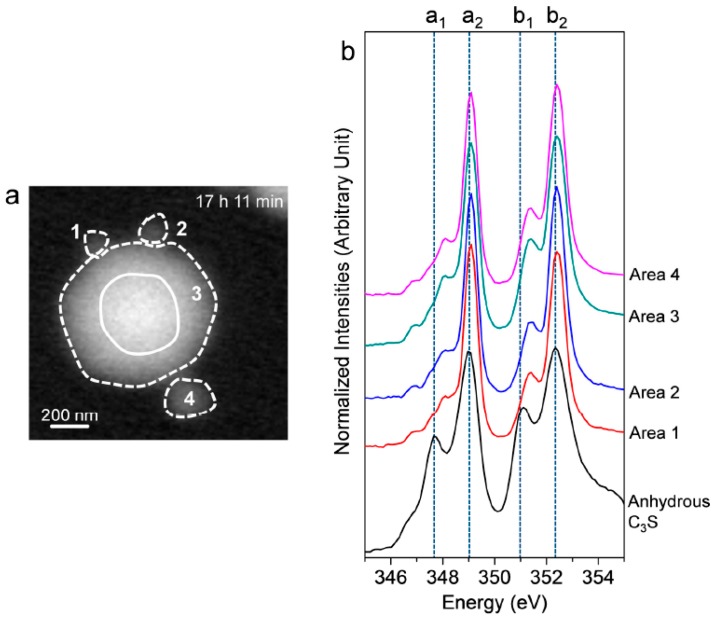
(**a**) STXM image contrast map of hydrated C_3_S after 17 h 11 min and (**b**) NEXAFS (near-edge X-ray absorption fine structure) spectra of the selected areas in (**a**). The center of the C_3_S particle, which is marked as a line circle, was excluded due to its thickness.

**Figure 6:**
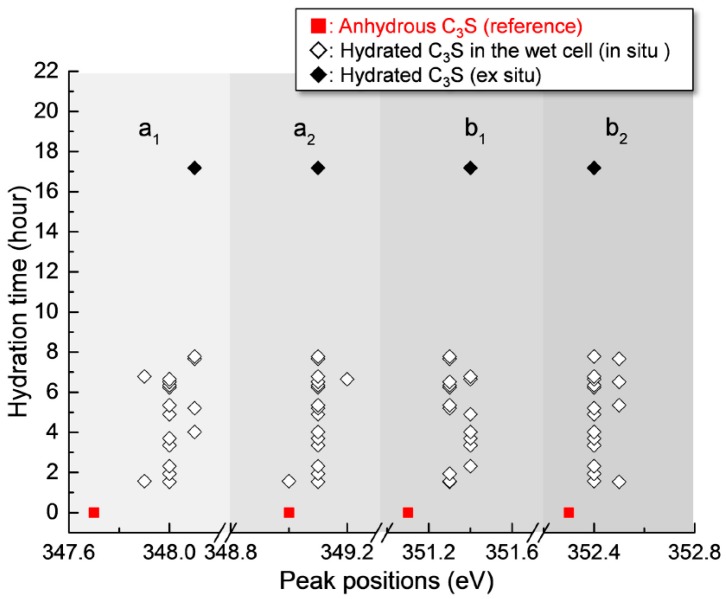
Peak positions of the Ca L_III,II_ edge NEXAFS spectra of the measured areas of hydrated C_3_S from 1 h 32 min to 17 h 11 min.

After 17 h 11 min, a different C_3_S particle in the same solution was analyzed (see Figure 5). To obtain overall spectroscopic information of the entire particle, an image stack was acquired in the range of 340 eV to 360 eV. The core of the particle, which is marked as a lined circle, was excluded from Ca analysis due to X-ray photon saturation. There was a very uniform distribution of Ca in hydrated C_3_S after 17 h 11 min (Figure 5a) compared to the image contrast map after 1 h 22 min (Figure 3a). The Ca L_III,II_ edge NEXAFS spectra of each analyzed area were identical in terms of shape and peak positions. The Ca L_III,II_ edge NEXAFS peak positions changed with hydration time from 1 h 32 min to 17 h 11 min, as illustrated in Figure 6. The Ca L_III,II_ edge peaks (a_1_, a_2_, b_1_, and b_2_) of the selected areas in hydrated C_3_S showed very similar values from 1 h 22 min to 17 h 11 min, with ±0.1 eV variation. On the basis of in situ and ex situ Ca L_III,II_ edge image contrast maps and the NEXAFS analysis, it may be concluded that the environment surrounding Ca in hydrated C–S–H on the surface of C_3_S in the wet cell changes quickly after contact with water in the dissolution process. Additionally, the Ca binding structure of the hydrated C–S–H is shown to be uniform and without significant changes. Furthermore, silicate polymerization during the acceleration period of C_3_S hydration, which resulted in the decrease of the Ca/Si ratio of C–S–H and a more connected environmental network (Q^n^) in the C–S–H sheet, had no significant influence on the Ca binding energy of C–S–H.

#### 3.2.3. Morphological Observations of the C_3_S Hydration Process (Si K Edge)

**Figure 7:**
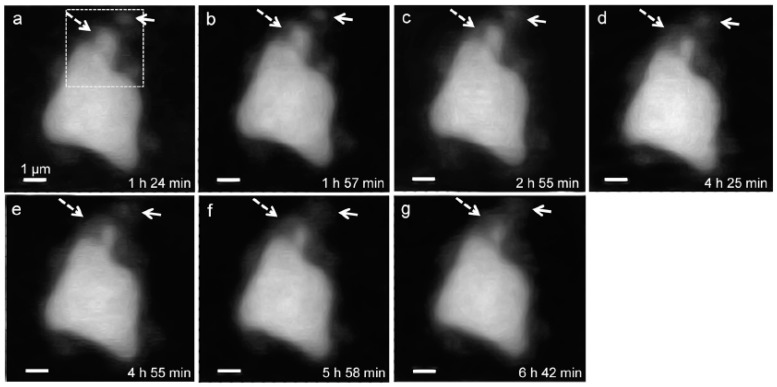
Morphology observations of a C_3_S particle in a wet cell using in situ STXM from 1 h 24 min to 6 h 42 min. Si elemental mapping was conducted by subtracting the image taken below the Si K edge from that taken above the edge. The dashed arrows indicate the dissolving part of the C_3_S particle, and the lined arrows point to the part of C–S–H formed on C_3_S. The dotted square area was magnified, as shown in Figure 8.

**Figure 8:**
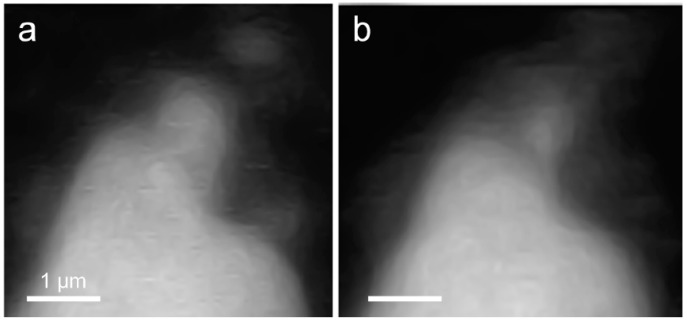
Comparison of morphological changes of a C_3_S particle in a wet cell using in situ STXM between (**a**) 1 h 24 min and (**b**) 6 h 42 min.

In situ STXM image contrast maps of Si of hydrated C_3_S in the wet cell, from 1 h 24 min to 6 h 42 min after mixing with water, are shown in Figure 7. Compared to the C_3_S particle observed in Ca L_III,II_ edge X-ray absorption imaging and NEXAFS, a larger particle was selected to obtain a better X-ray absorption contrast. This is because Si K edge X-ray absorption imaging requires a higher energy for the Si K edge (~1860 eV). Elemental mapping of Si was carried out by subtracting an image taken below the Si K edge from one taken above the pre-edge, as done for Ca. The dashed and lined arrows in Figure 7 indicate the dissolving part of the C_3_S particle and a part of the hydration products assumed to be C–S–H, respectively. From 1 h 24 min (Figure 7a) to 6 h 42 min (Figure 7g), a part of the C_3_S particle was dissolved by the hydration process. Furthermore, the interface of the C_3_S particle changed and became hazy as a result of the formation of C–S–H, and the area of C–S–H formed at the interface of the C_3_S particle increased over time. The selected area, indicated as a square in Figure 7a, was magnified in order to confirm the morphological changes occurring during the hydration process. Contrast maps of the magnified Si K edge images acquired at 1 h 24 min and 6 h 42 min are shown in Figure 8. Morphological changes can be observed only in localized areas whereas no significant changes were found in other areas of C_3_S. It is worth noting that this localized C_3_S dissolution was already discussed in the results of the in situ Ca L_III,II_ edge image contrast maps. C–S–H precipitation also mainly occurred in regions of comparatively larger surface areas on C_3_S. Images at the Ca L_III,II_ edge (~340 eV) showed a localized Ca-dissolution process from C_3_S and nano C–S–H seeded in the solution. On the other hand, imaging at the Si K edge appeared to be more suitable for observing the C–S–H formation process occurring by silicate polymerization. Details of the effect of silicate polymerization on the binding structure of C–S–H formed on C_3_S are discussed in the following section.

#### 3.2.4. Si K Edge NEXAFS Analysis

**Figure 9:**
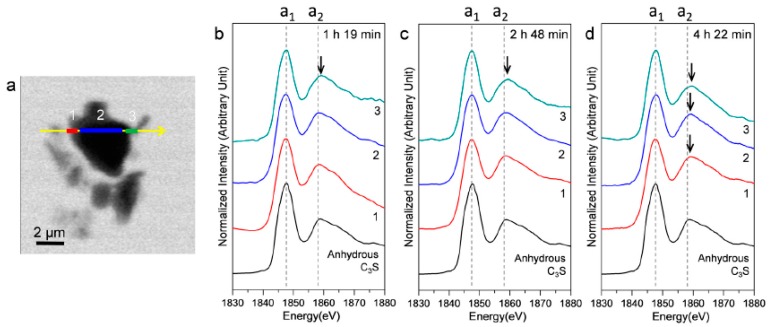
STXM image and in situ Si K edge NEXAFS spectra of selected areas in the line scan of a C_3_S particle in the wet cell. (**a**) STXM images of the C_3_S sample in a wet cell taken at 1840 eV after 1 h 19 min and the Si K edge NEXAFS of the selected area of the line scan after (**b**) 1 h 19 min; (**c**) 2 h 48 min; and (**d**) 4 h 22 min.

Figure 9 shows an STXM image taken at 1840 eV, in addition to the Si K edge NEXAFS spectra of three selected linescans (indicated as 1, 2, and 3) in the hydrating C_3_S particle. In the Si K edge NEXAFS of the cement hydrates, peak a_1_, the main peak, is attributed to the Si K edge induced by the electronic transition from Si 1s to the anti-bonding t_2_ (3p-like state) orbital. The peak a_2_ is assigned to effects of multiple scattering beyond the second coordination sphere [35]. The peak positions of a_1_ and a_2_ and the energy separations between the two peak positions (a_2_−a_1_) are given in Table 2. Our previous study showed that the increase of the Si K edge binding energies of C–S–H correspond to a decrease in the Ca/Si ratio, resulting from silicate polymerization of C–S–H in the hydration process [10,25,36]. Increased polymerization of silicate tetrahedra results in a higher degree of Q^n^ in C–S–H and creates a higher effective charge on the Si atom [36]. In Figure 9, the right and left layers around C_3_S are presumed to be hydrating C–S–H layers on C_3_S. Peak a_1_, after 1 h 19 min, showed a smaller value (0.1–0.3 eV) than that of anhydrous C_3_S. On the other hand, peak a_2_ in area 3, after 1 h 19 m, was at a higher position, which resulted in a larger energy distance (0.4 eV) between peaks a_1_ and a_2_. The decrease in peak a_1_, measured at 1 h 19 min, is due to the increased pre-occurrence of silicate monomers in the dissolution of C_3_S before the induction period (~1 h) because Si in the dissolved SiO_4_^4−^ monomer has a lower binding energy than anhydrous C_3_S due to the low degree of Q^n^. After 2 h 48 min and 4 h 22 min, both peaks a_1_ and a_2_ in areas 1 and 3 (outer C–S–H layer) showed a tendency to increase. However, the peak shift of a_2_ was larger so that the distances between the two peaks showed values that were 0.5–0.6 eV larger compared to anhydrous C_3_S. Thus, the silicate polymerization process in C–S–H, resulting in enhanced Q^n^, has a greater influence than core Si atoms on multiple scattering beyond the second coordination sphere of Si. This is in good agreement with our previous study performed in dried conditions [25].

**Figure 10:**
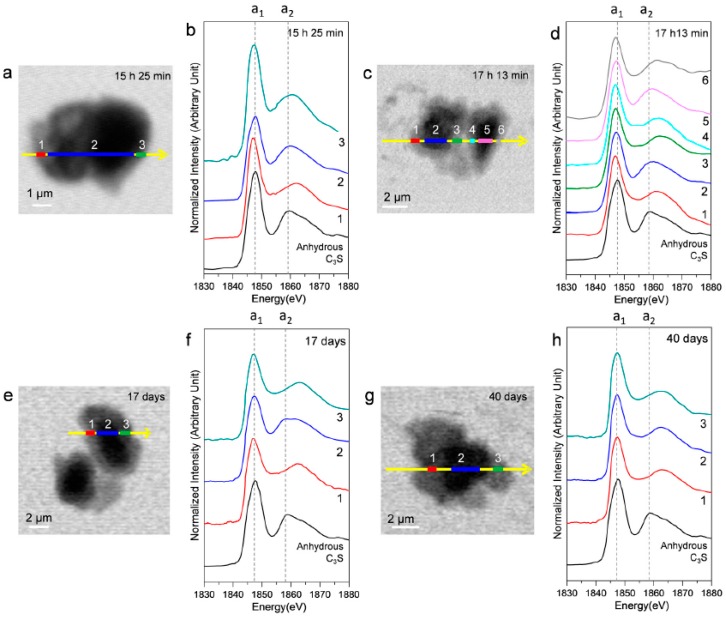
Ex situ STXM images and Si K edge NEXAFS spectra of the C_3_S sample in a wet cell after 15 h 25 min (**a**,**b**); 17 h 13 min (**c**,**d**); 17 days (**e**,**f**); and 40 days (**g**,**h**). Layers with different contrasts in the images were selected for NEXAFS analysis.

Ex situ STXM measurements were performed on a hydrated C_3_S paste at the end of the deceleration period, as indicated by the isothermal calorimetry curve. Figure 10 shows the STXM images taken at 1840 eV and the Si K edge NEXAFS spectra of the selected areas after 15 h 25 min, 17 h 13 min, 17 days, and 40 days. Linescans were measured at the core and the boundary of hydrated C_3_S. Note that X-ray photons transmitted through the core area contain the X-ray absorption information of the C–S–H layer and anhydrous C_3_S. The thickness of the C–S–H layer, which exhibited varying contrasts in the Si image contrast map, increased over time. This indicated the progress of C–S–H formation. The resulting Si K edge NEXAFS peak positions are given in Table 3. The peak a_1_ value in all the spectra showed a variance of +0.3–(−0.6) eV as compared to anhydrous C_3_S, without any obvious trend of variation. On the other hand, peak a_2_ shifted more than peak a_1_, and the distance between these two peaks increased significantly with the progress of hydration. Interestingly, at 15 h 25 min, the energy separations between the peaks in the core area (area 2) had not changed as compared to the result after 4 h 22 min (11.7 eV). However, the C–S–H layer (areas 1 and 3) showed values of 1.8 eV and 3.4 eV, larger than those in the core area (area 2), indicating the progress of hydration in the outer C–S–H layer on C_3_S. At 40 days of hydration, C–S–H layers in C_3_S and the core area exhibited identical Si K edge NEXAFS. No trace of anhydrous C_3_S was found in the core area, indicating complete hydration of C_3_S. As found in the results of in situ STXM performed at an early age, silicate polymerization occurring during the C–S–H precipitation has a smaller effect on the chemical shift of peak a_1_ as compared to peak a_2_.

**Figure 11:**
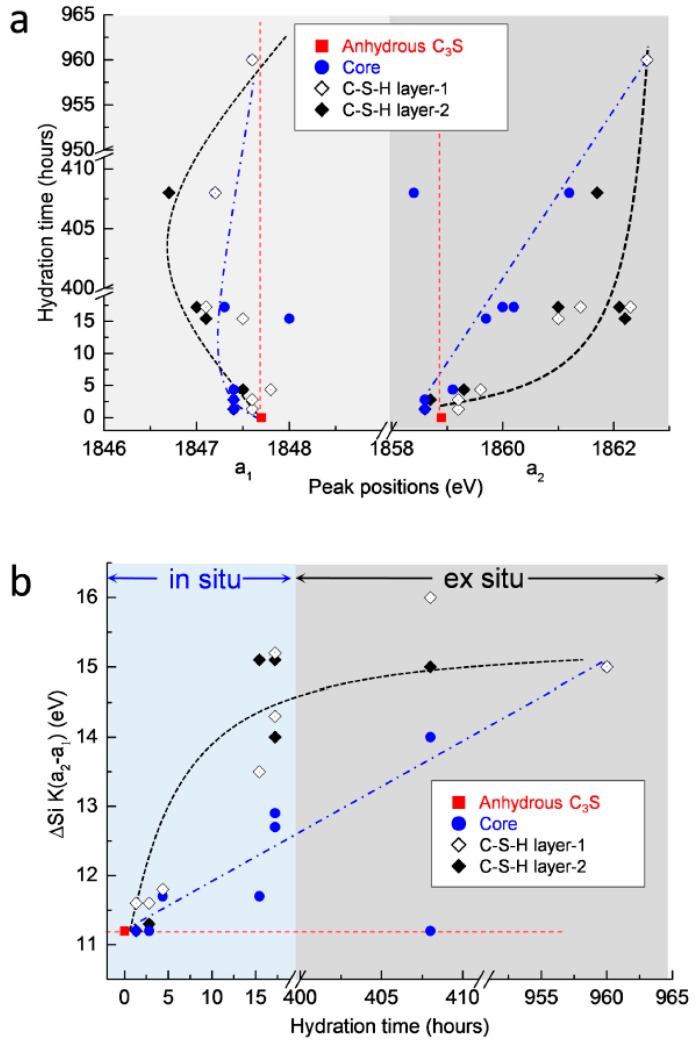
(**a**) Changes in peak positions (a_1_ and a_2_) of the Si K edge NEXAFS and (**b**) difference in energy corresponding to changes in peak position (a_2_−a_1_) during the C_3_S hydration process.

The measured peak positions and energy separations between the two peaks of the Si K edge after 1 h 19 min to 40 days of hydration are plotted in Figure 11. In terms of the position of peak a_1_, shown in Figure 11a, there were no significant changes in the core area of C_3_S. On the other hand, peak a_1_ in the C–S–H formed on the surface of C_3_S increased slightly until 4 h 22 min, then decreased at 17 days, but then increased again at 40 days. A possible explanation for these variations is that the C_3_S particles selected for in situ and ex situ measurements may have had different original peak positions compared to the reference C_3_S. However, effects generated from the differences of the particles can be excluded. This is because anhydrous C_3_S was stored in N_2_ to minimize carbonation and showed identical Si K edge NEXAFS spectra when measured for different particles several times. Therefore, with regard to the chemical shifts of peak a_1_, the following assumptions can be made. Note that in the in situ measurements, the change of the peak position in the Si K edge NEXAFS was measured in a small area on the surface of C_3_S. The decreased value of peak a_1_ in the Si K edge found at 1 h 19 min, compared to anhydrous C_3_S, was due to the pre-dissolved silicate monomers present before the induction period (1 h 19 min). Therefore, the increase in energy of peak a_1_ after 1 h 19 min could be induced by silicate polymerization occurring during the formation of outer C–S–H. On the other hand, in the ex situ measurements from 15 h until 17 days, the linescan of C–S–H layers contained X-ray absorptions of both early-forming outer C–S–H and later-forming inner C–S–H. The dissolution and precipitation processes, which simultaneously occurred during the inner C–S–H formation slowly from 15 h until 17 days, may have induced the shift of peak a_1_ to lower energy and to higher energy, respectively. From 15 h until 17 days of hydration, the dissolution prevailed over the precipitation in the C–S–H layer. This led to the shift of peak a_1_ to a lower energy compared to anhydrous C_3_S. On the other hand, from 17 days to 40 days, silicate polymerization occurring during C–S–H precipitation was dominant over the dissolution process. This resulted in shift of peak a_1_ to higher energy of the Si K edge NEXAFS. However, due to the fact that the enhancement of dissolution in C_3_S could be expected in the dilute system of the current study, it is still required to augment these measurement gaps between 17 days and 40 days to verify the effect of dissolution and precipitation on the shift of peak a_1_. Moreover, continuous in situ Si K edge NEXAFS measurements on the same particle, from the initiation of hydration to the end of the acceleration period, are still necessary to verify the assumptions described above.

With regard to peak a_2_, both the core area and the C–S–H layer showed increases from the initiation of hydration, over time. While the peak position in the core area increased gradually, the position of the C–S–H layer showed a more rapid shift to a higher energy at an early age (until 17 h), and then increased gradually. This supports the idea that the formation of C–S–H occurs from the outer areas of the C_3_S particles and that the C–S–H layer formed at an early age slows down the hydration of C_3_S into the core area. Although modifications in microstructure and local binding during C_3_S hydration obviously justify the utility of the wet cell for in situ STXM investigations, the following issues should be considered to expand the measurement range of C_3_S hydration: (a) a new type of wet cell is required to measure the beginning of hydration because the wet cell used here must be prepared at least 1 h before initiating the measurement; (b) hydration retardants can be added into the dilute suspensions to slow down the dissolution of C_3_S, which could facilitate better observation of chemical shifts in X-ray absorption NEXAFS, occurring with C_3_S hydration before and during the induction period. An extended theoretical approach is also needed, first to study the dissolution and precipitation of cement clinker, and also to verify the spectroscopic results reported in the current study. This theoretical approach may be based on molecular orbital or multiple scattering theory. In situ STXM will likely open up new opportunities to study phase hydration kinetics in other PC clinkers, such as belite, and also to study the effect of mineral and chemical admixtures on hydration at an early age.

## 4. Conclusions

The present work represents the first use of in situ soft X-ray spectromicroscopy to observe C_3_S hydration in wet conditions from induction to deceleration. Ca L_III,II_ and Si K edge X-ray absorption imaging and NEXAFS analysis were conducted. In situ image contrast mapping of Ca was used to visualize the formation of nano C–S–H seeds in the C_3_S solution at an early age. Then, the development of a fibrillar C–S–H morphology in the outer areas of C_3_S was also observed. In situ Ca L_III,II_ and Si K edge X-ray absorption images indicate that localized dissolution and precipitation processes occur at the edge of C_3_S or in regions with a comparatively larger surface area. Dissolution of C_3_S in the induction period immediately changes the Ca binding energy of the surface area of C_3_S. However, no significant changes from the acceleration period are observed in terms of the Ca binding energy of C–S–H. This indicates that the dissolution of C_3_S affects the Ca binding energy. The silicate polymerization process has a minor effect on the Ca binding energy in CaO layers of C–S–H. It is also found that the C–S–H layer formed on the outer areas of C_3_S in the acceleration period retards hydration of the core area of C_3_S. Silicate polymerization in C–S–H contributes to the multiple-scattering effects in Si K edge NEXFAS compared to the binding energy of Si at the main edge. The change of peak positions of the Si K edge could provide a spectroscopic basis for estimating the degree of silicate polymerization in C–S–H.

## Figures and Tables

**Table 1:** Peak positions and energy separations between the peak positions of the Ca L_III,II_ edge NEXAFS (near-edge X-ray absorption fine structure) spectra of the line scan of C_3_S in a wet cell from 1 h 32 min to 7 h 74 min. Ex situ Ca L_III,II_ edge data of the image stack of C_3_S measured at 17 h 11 min are also presented.

Sample	Peak Positions (eV)				Splitting of L_III_ and L_II_ (eV)	
	a_1_	a_2_	b_1_	b_2_	∆L_III_ (a_2_−a_1_)	∆L_II_ (b_2_−b_1_)
Anhydrous C_3_S [25]	347.7	349	351.1	352.3	1.3	1.2
Ca(OH)_2_ [25]	347.6	349.1	351	352.4	1.5	1.4
1 h 32 min	348	349.1	351.3	352.5	1.1	1.2
1 h 34 min	347.9	349	351.3	352.4	1.1	1.1
1 h 56 min	348	349.1	351.3	352.4	1.1	1.1
2 h 19 min	348	349.1	351.4	352.4	1.1	1
3 h 21 min	348	349.1	351.4	352.4	1	1
3 h 42 min	348	349.1	351.4	352.4	1.1	1
4 h 01 min	348.1	349.1	351.4	352.4	1	1
4 h 54 min	348	349.1	351.4	352.4	1.1	1
5 h 12 min	348.1	349.1	351.3	352.4	1	1.1
5 h 21 min	348	349.1	351.3	352.5	1.1	1.2
6 h 15 min	348	349.1	351.3	352.4	1	1.1
6 h 21 min	348	349.1	351.3	352.4	1	1.1
6 h 31 min	348	349.1	351.3	352.5	1.1	1.2
6 h 39 min	348	349.2	351.4	352.4	1.2	1
6 h 47 min	347.9	349.1	351.4	352.4	1.2	1
7 h 40 min	348.1	349.1	351.3	352.5	1	1.2
7 h 47 min	348.1	349.1	351.3	352.4	1	1.1
17 h 11 min (Ex Situ)	348.1	349.1	351.4	352.4	1	1

**Table 2:** Peak positions and energy separations between peak positions of the Si K edge NEXAFS line scan spectra of C_3_S in the wet cell after 1 h 19 min, 2 h 48 min, and 4 h 22 min.

Sample	Scan Areas	Peak a_1_ (eV)	Peak a_2_ (eV)	Δa_2_−a_1_ (eV)
Anhydrous C_3_S [25]	-	1847.7	1858.9	11.2
1 h 19 min (In Situ)	1	1847.4	1858.6	11.2
	2	1847.4	1858.6	11.2
	3	1847.6	1859.2	11.6
2 h 48 min (In Situ)	1	1847.4	1858.7	11.3
	2	1847.4	1858.6	11.2
	3	1847.6	1859.2	11.6
4 h 22 min (In Situ)	1	1847.5	1859.3	11.8
	2	1847.4	1859.1	11.7
	3	1847.8	1859.6	11.8

**Table 3:** Peak positions and energy separations between peak positions of the Si-K edge NEXAFS line scan spectra of C_3_S after 15 h 25 m, 17 h 13 m, 17 days, and 40 days.

Sample	Scan Areas	Peak a_1_ (eV)	Peak a_2_ (eV)	Δa_2_−a_1_ (eV)
Anhydrous C_3_S [25]	-	1847.7	1858.9	11.2
15 h 25 min (Ex Situ)	1	1847.1	1862.2	15.1
	2	1848	1859.7	11.7
	3	1847.5	1861	13.5
17 h 13 min (Ex Situ)	1	1847	1861	14
	2	1847.3	1860.2	12.9
	3	1847.1	1862.3	15.2
	4	1847	1862.1	15.1
	5	1847.3	1860	12.7
	6	1847.1	1861.4	14.3
17 days (Ex Situ)	1	1847.2	1862.2	15
	2	1847.2	1858.4, 1861.2	11.2, 14
	3	1847.2	1863.2	16
	1	1847.6	1862.6	15
40 days (Ex Situ)	2	1847.6	1862.6	15
	3	1847.6	1862.6	15
